# The impact of expert visual guidance on trainee visual search strategy, visual attention and motor skills

**DOI:** 10.3389/fnhum.2015.00526

**Published:** 2015-10-14

**Authors:** Daniel R. Leff, David R. C. James, Felipe Orihuela-Espina, Ka-Wai Kwok, Loi Wah Sun, George Mylonas, Thanos Athanasiou, Ara W. Darzi, Guang-Zhong Yang

**Affiliations:** ^1^Hamlyn Centre for Robotic Surgery, Imperial College LondonLondon, UK; ^2^National Institute for Astrophysics, Optics and Electronics (INAOE)Tonantzintla, Mexico

**Keywords:** functional near infrared spectroscopy, optical topography, neuroergonomics, graph theory, collaborative gaze, visual attention, skills assessment, mentoring

## Abstract

Minimally invasive and robotic surgery changes the capacity for surgical mentors to guide their trainees with the control customary to open surgery. This neuroergonomic study aims to assess a “Collaborative Gaze Channel” (CGC); which detects trainer gaze-behavior and displays the point of regard to the trainee. A randomized crossover study was conducted in which twenty subjects performed a simulated robotic surgical task necessitating collaboration either with verbal (control condition) or visual guidance with CGC (study condition). Trainee occipito-parietal (O-P) cortical function was assessed with optical topography (OT) and gaze-behavior was evaluated using video-oculography. Performance during gaze-assistance was significantly superior [biopsy number: (mean ± SD): control = 5.6 ± 1.8 vs. CGC = 6.6 ± 2.0; *p* < 0.05] and was associated with significantly lower O-P cortical activity [ΔHbO_2_ mMol × cm [median (IQR)] control = 2.5 (12.0) vs. CGC 0.63 (11.2), *p* < 0.001]. A random effect model (REM) confirmed the association between guidance mode and O-P excitation. Network cost and global efficiency were not significantly influenced by guidance mode. A gaze channel enhances performance, modulates visual search, and alleviates the burden in brain centers subserving visual attention and does not induce changes in the trainee’s O-P functional network observable with the current OT technique. The results imply that through visual guidance, attentional resources may be liberated, potentially improving the capability of trainees to attend to other safety critical events during the procedure.

## Highlights

1. A randomized crossover study assessing the impact of trainer visual guidance upon trainee visual cognition, occipito-parietal (O-P) brain function and technical performance.
2. Visual guidance is associated with enhanced gaze behavior, improved technical accuracy and attenuated activity across O-P cortices.
3. Parameters of network performance such as cost and global efficiency are not detrimentally effected by visual guidance.

## Introduction

In high-risk industry, collaboration between operators is integral to performing goal-orientated tasks successfully (e.g., pilots, air-traffic controller, surgeons, etc). Regarding surgery, collaboration is necessary between surgeons and their assistant(s), theatre nurse(s) and occasionally members of allied specialties. Recent developments in technologies for robotic surgery such as dual console systems (e.g., da Vinci^®^ Si) enable two surgeons to operate simultaneously, facilitating both high-level co-operation and mentorship as well as potentially streamlining the operators’ cognitive resources towards improved safety. However, in this scenario, it is important that communication between both surgeons is effective to enable a seamless flow of information between the two operators and ensure an efficient workflow. Similarly, excellent communication facilitates technical skills training in surgery. During “open” surgery, expert trainers’ employ a variety of methods for communication with trainees that include a combination of verbal instruction, physical pointing or actual demonstration(s). However, during robotic minimally invasive surgery (MIS), there may be circumstances in which the trainee or collaborating surgeon is using both instruments simultaneously within the operative field of view, constraining the trainer/master surgeon and rendering them reliant solely on verbal communication.

Within MIS and robotic surgery, techniques exist such as telestration that aid information transfer between surgeons and/or between trainer and trainee. Telestration allows information to be “*drawn*” onto a monitor at a remote site by the surgeon guiding the procedure. This information is then displayed on the operator’s screen with the aim of guiding performance and may be undertaken either remotely or locally (Ferguson and Stack, 2010). Remote guidance or telementoring enables surgeons to be guided by a mentor at a location remote from the operation. This form of instruction has been applied to better enable regional experts to guide surgeons at local centers and to provide assistance and mentoring from surgical experts in other countries (Micali et al., 2000; Schlachta et al., 2010).

There has been interest in the role that gaze behavior may have in improving the flow of communication between collaborating subjects. For example, it has been demonstrated that shared gaze during visual collaboration enables a more efficient search strategy when compared to verbal collaboration alone (Brennan et al., 2008). Therefore, it is anticipated that observing a guiding surgeon’s point of regard instead of, or in conjunction with their verbal instruction(s) will significantly improve the performance of the operating surgeon by providing supplementary cues critical to task success. Based on this concept, a new system referred to as “*collaborative gaze control*” (CGC) was developed to enable an operating surgeon to be directed by visual guidance as opposed to or in conjunction with verbal instruction(s) from an expert (Kwok et al., 2012). With CGC enabled, the trainer’s gaze behavior is extracted in real-time. Their point of regard is subsequently relayed to the trainee’s screen, which may be in a remote location. Therefore, the trainee’s operative manoeuvres can be directed more precisely, potentially obviating the dependence on verbal instruction(s). Importantly, in manipulating target salience, visual search is modulated leading to enhanced behavioral performance (Avraham et al., 2008).

More recently, there is evidence that workload can be inferred from saccadic eye movements (Tokuda et al., 2011), pupillary responses (Zheng et al., 2015) and blink frequency (Zheng et al., 2012). Challenging, effortful visual search results in greater visual cortical (V1) excitation (Kojima and Suzuki, 2010). Evaluating the impact that technological manipulation of visual search has on an operator’s cortical function helps to determine whether performance enhancement is offset by the need for greater attentional demands at brain level. This is encompassed by “*neuroergonomics*” which concerns the investigation of the brain behavior at work (Parasuraman, 2003), a paradigm that has been applied to surgery in order to investigate how recruited brain regions may be modulated by novel performance-enhancing tools (James et al., 2010b, 2013).

In order to examine this effect, functional Near Infrared Spectroscopy (fNIRS) a non-invasive neuroimaging modality is utilized to measure task-evoked fluctuations in oxygenated and deoxygenated hemoglobin (HbO_2_ and HHb respectively) within cortical tissues that reflects the magnitude of cortical activation (Jöbsis, 1977). This is based upon the principle that neuronal activity and the associated increased metabolic demand within the brain leads to local hemodynamic changes, so termed *“neurovascular coupling”* (Roy and Sherrington, 1890). Unlike functional magnetic resonance imaging (fMRI), fNIRS is relatively resistant to motion artifact and can be used in conjunction with ferromagnetic instruments and has been successfully applied to monitor the cortical responses in surgeons (Leff et al., 2008a,b,c; Ohuchida et al., 2009; James et al., 2011, 2013). Broadly, these studies highlight the importance of the prefrontal cortex (PFC) in supporting “cognitive phases” of skill learning (Leff et al., 2008a), evolution in PFC excitation with technical skills training (Leff et al., 2008c), and relative PFC redundancy amongst expert surgeons (Ohuchida et al., 2009). More recently, investigators have demonstrated the impact of the type of learning (e.g., implicit vs. explicit) and the influence of technology to stabilize performance and enhance neuronal efficiency amongst surgeons (Zhu et al., 2011; James et al., 2013).

Functional brain connectivity captured in coherence or cross-correlation between different brain regions can be used to investigate efficiency in brain networks (Zhu et al., 2011; James et al., 2013). Graph Theory, a popular method for interrogating brain networks, can model the organization, development and function of complex networks (Sporns et al., 2004; Bullmore and Sporns, 2009; Sporns, 2011) and has been successfully employed to networks derived from fNIRS data (Niu et al., 2012; James et al., 2013). In this regard, studies investigating graph topology such as the number of connections, cost and efficiency have demonstrated associations between task performance and brain network efficiency or cost-efficiency (Bassett et al., 2009). Despite the above, there have been no studies investigating the influence of varying trainer/mentor guidance on brain function or network architectures amongst trainees.

The aim of this paper is to investigate the influence of a gaze channel on changes in visual search strategies, technical performance, and brain behavior in a group of task naïve subjects being instructed to perform simulated biopsy using robotic MIS. Therefore, it is anticipated that compared to verbal guidance technical procedural skills may be superior during gaze-assistance owing to the improved perceptual flow of information to the trainee. The primary hypothesis is that increased target saliency will lead to a *“bottom-up”* search strategy, reflected in a more focused pattern of V1 activation and a reduction in the need for recruitment of extra-striatal visual association areas. Conversely, verbal communication (gold standard) is anticipated to lead to a more effortful *“top down”* visual search strategy, necessitating recruitment of additional cortical regions outside V1, manifest as greater excitation in centers of visual attention. The secondary hypothesis is that collaborative gaze may facilitate the flow of information transfer in the visual-parietal network manifest as reduced network costs, improved efficiency and reduced network burden.

## Materials and Methods

### Subjects

The study was carried out in accordance with the recommendations of the Local Regional Research Ethics Committee (LREC 05/Q0403/142) with written informed consent from all subjects. All subjects gave written informed consent in accordance with the Declaration of Helsinki. Following ethical approval a randomized control trial was conducted in which 20 subjects (1 female) were recruited from Imperial College London (mean age, years ± SD = 28.9 ± 1.5). Left-handed subjects and those with a history of neuropsychiatric illness or previous exposure to the task were excluded (Orihuela-Espina et al., 2010). Subjects were included on the basis that they were task naïve. The task was performed under both guidance conditions (order randomized) such that subjects served as their own controls and bias associated with learning or ordering effects was minimized.

### Task Paradigm

**Figure 1:**
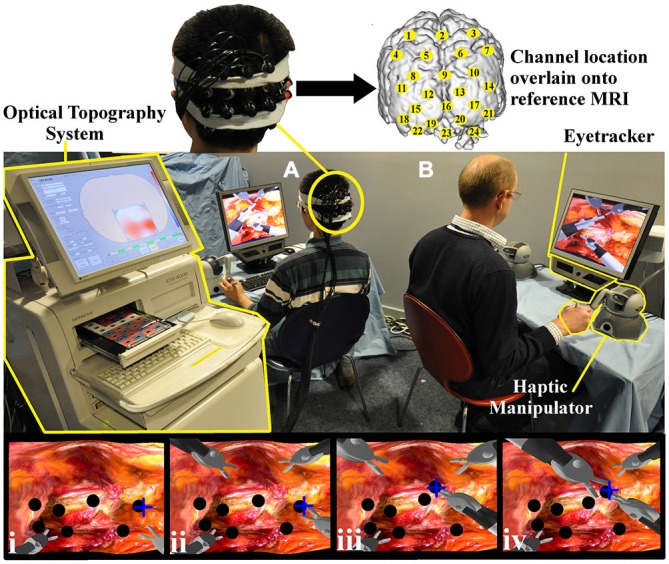
**Experimental task set up.** Both the trainee **(A)** and trainer **(B)** control the virtual instruments, each with two haptic manipulators (Phantom Omni, SensAble Tech, USA). The trainer’s right hand manipulator is highlighted (yellow). Gaze behavior is detected with portable eyetracker (X50 eyetracker, Tobii Technologies, Sweden) situated below both monitors (trainer eyetracker highlighted yellow). An Optical topography (OT) system (ETG-4000, Hitachi Medical Corp. Japan) positioned outside the trainee’s field of view (left, highlighted) records cortical hemodynamic data from 24 cortical loci (channels). Appropriate channel locations (yellow circles) are understood by projecting 3D positional data onto a T1 weighted MRI image (upper subplot). The lowermost row of channels was centered on Oz of the International 10–10 system (Jurcak et al., 2007). Task images can be appreciated on trainer and trainee monitors and sample screen shots are represented in which the trainee’s instruments are located inferiorly (i–iv). With the collaborative gaze channel (CGC) enabled, the trainee regards the blue cross indicating the intended biopsy target (i). The trainee then grasps the nodule (black circle) (ii) and passes it to the trainer’s instrument (iii–iv). With the channel disabled, the trainee performs identical maneouvres but only with verbal instructions from the trainer.

The robotic surgical task entailed the subject (*“trainee”*) and an expert (*“trainer”*) collaborating in taking virtual biopsies from a simulated gastric mucosa in a shared surgical environment as depicted in Figure 1. Haptic manipulators (Phantom, Omni, SensAble Technologies, USA) were used to control robotic graspers in the virtual scene. The task necessitated the trainee take a virtual biopsy and pass the specimen to the guiding trainer. Both the trainee’s and the trainer’s graspers were visible within the same field of view with the former located inferiorly and the latter superiorly as depicted in Figure 1 (panels i–iv). Within the operative field, seven nodules were visible to the trainee. The choice of nodule for biopsy was randomly determined and this selection was available only to the trainer. Therefore, the appropriate biopsy site had to be conveyed to the trainee either visually or verbally by the trainer. Once the biopsy was taken by the trainee, the specimen was passed towards the trainer’s graspers and when successfully transferred to the trainer, it disappeared from the field of view. This process was repeated as many times as possible during the allotted task periods.

Prior to commencing the study, all subjects received a standardized period of task familiarization. All subjects performed the simulated biopsy task under verbal (control) and visual instruction (CGC; Kwok et al., 2012). The order was randomized (random number generator) in order to control for learning effects. Regarding the control task, the location of the biopsy site was described by the trainer using verbal instructions. With CGC enabled, a portable eyetracker (×50 eyetracker Tobii Technologies, Sweden) situated beneath the trainer’s monitor detected their fixation point and conveyed this to the trainee’s screen as a cross. Therefore, with CGC enabled, the trainer’s target selection would be conveyed to the trainee. For each condition (verbal and CGC) a block design experiment was employed comprising a baseline rest period (30 s) followed by five task blocks each of which comprised alternating episodes of simulated nodule biopsy (30 s) and inter-trial rest periods (30 s). During rest periods, subjects were asked to remain still with their eyes open regarding a black screen on the task monitor. Within functional neuroimaging experiments, block design paradigms have the advantage of allowing the hemodynamic response to return to baseline between each session, therefore providing reliable indices of task-evoked cortical activity. Furthermore, the block design allows task data to be averaged, increasing the signal to noise ratio.

### Cortical Activity

Brain activation was assessed using a commercially available 24-channel Optical topography (OT) system (ETG-4000, Hitachi Medical Corp., Japan). Sixteen optodes (8 emitters and 8 detectors) were positioned in a 4 × 4 array over the O-P cortices as displayed in Figure 1. A “channel” represents a banana-shaped volume of cortex where changes in absorption of near infrared light from the optode emitters are interpreted as changes in HbO_2_ and HHb. The array was centered on “Oz” of the International 10–20 system (Jurcak et al., 2007) with the intention of capturing activation within the visual cortex. Cortical data was subject to both manual and automated data integrity checks (Orihuela-Espina et al., 2010) to identify and eliminate data contaminated with noise, optode movement and saturation-related artifacts (i.e., apparent non-recordings and “mirroring”). Since both ambient light and near infra-red light from eye-tracking systems have the potential to influence OT data (Orihuela-Espina et al., 2010), laboratory lights were dimmed and the probes were shielded using a combination of external fixation tapes and shower cap.

### Technical Performance

The number of nodules that the trainee was able to successfully biopsy and transfer to the trainer’s graspers across the task period and the trainee’s instrument pathlength (metres) were recorded and used as objective metrics of technical performance. This was preferred to restricting the overall number of moves towards calculating time/nodule biopsied, and helped to ensure that subjects were focusing on the task quality and not the procedural time, or perceiving the number of movements.

### Gaze Behavior

Subject and trainer gaze behavior was recorded throughout the study with portable eyetracking technology (×50 eyetracker, Tobii Technologies, Sweden) situated beneath the task monitor (as displayed in Figure 1). The gaze behavior of the trainer was interrogated to derive their fixation point in order to display this as a cross on the trainee’s monitor thereby facilitating gaze-guidance in CGC (study condition). The trainer’s fixation point was not visible to the trainee during episodes of verbal guidance (control condition). The trainee’s fixation points were recorded to determine the time taken, termed “gaze latency” (GL, seconds), to fixate on the same area of the surgical scene as the expert.

### Heart Rate Monitoring

A portable band electrocardiogram (Bioharness v2.3.0.5; Zephyr Technology Limited, USA) was used to acquire continuous heart rate data, from which heart rate variability (HRV) was derived and used to infer subject stress (Task Force of the European Society of Cardiology the North American Society of Pacing Electrophysiology, 1996).

## Data Analysis

### Cortical Hemodynamics

Cortical hemodynamic data and network graph econometrics were observed to be non-Gaussian and therefore analyzed using non-parametric tests of significance. Channel-wise cortical activation was determined as a task-evoked statistically significant increase in HbO_2_ coupled to a significant decrease in HHb from baseline rest (Wilcoxon Rank Sign, *p* < 0.05). For each channel of data and hemoglobin species a variable ΔHb was computed (Hb task–Hb rest). To investigate the influence of the mode of guidance (CGC vs. control) and stress on cortical hemodynamics (i.e., ΔHbO_2_ and ΔHHb) random effects models (REM) were generated (Intercooled Stata, v10.0 for windows, Stata Corporation, USA).

Cortical hemodynamic data was subsequently used to construct a task-evoked network of the 24 channels using graph theory (Bullmore and Sporns, 2009). A 24 × 24 bidimensional cross-correlation matrix was constructed by cross-correlating data between all channels, as previously described (James et al., 2013). This matrix represents the strength of functional associations within the network of 24 channels. Comparisons between graphs of different functional networks are potentially sensitive to the method used for thresholding, for which an optimal solution does not yet exist (van Wijk et al., 2010). Therefore, to evaluate the active network, the matrix was pruned to eliminate “inactive” graph nodes. This approach renders a network for each subject during each task condition.

Econometric data from these networks was then calculated to derive: (a) the number of network connections; (b) the maximum global efficiency (Achard and Bullmore, 2007); (c) the normalized cost (Achard and Bullmore, 2007); and (d) the task-induced *“network burden”* (James et al., 2010a). Network economy is defined as efficiency minus cost (Achard and Bullmore, 2007). The network burden is defined here as—economy which equates to “cost-efficiency”. If a network is economical the cost-efficiency is high and accordingly the network burden is low. Network measures were also compared between the study and control groups using REM analysis to determine whether the mode of guidance (CGC vs. control) significantly influenced network econometrics. Statistical significance was set at *p* = 0.05.

### Performance and Gaze Behavior

The number of nodules biopsied by each subject during the allotted task time and the instrument pathlength (metres) were determined. GL (seconds) was derived from the eye-tracking data stream. Behavioral performance and GL data was observed to be Gaussian and therefore analyzed using paired *t*-tests. These data were subsequently incorporated into the REM analysis in order to assess whether the guidance mode (control vs. CGC) was a predictor of performance accuracy and efficiency in visual search.

### Heart Rate Analysis

HRV as calculated by the standard deviation of the R to R interval (SD_RR_) was derived from the HR data stream (Task Force of the European Society of Cardiology the North American Society of Pacing Electrophysiology, 1996). The SD_RR_ decreases under stress and was incorporated into the REM analysis, to exclude any potential confounding effect that differences in HRV or changes in mean HR may exert on changes in cortical hemodynamics. Furthermore, HRV was utilized to determine which mode of guidance (verbal vs. CGC) trainee’s found the most stressful by undertaking a univariate random effects analysis (*p* = 0.05).

## Results

### Technical Performance

**Figure 2:**
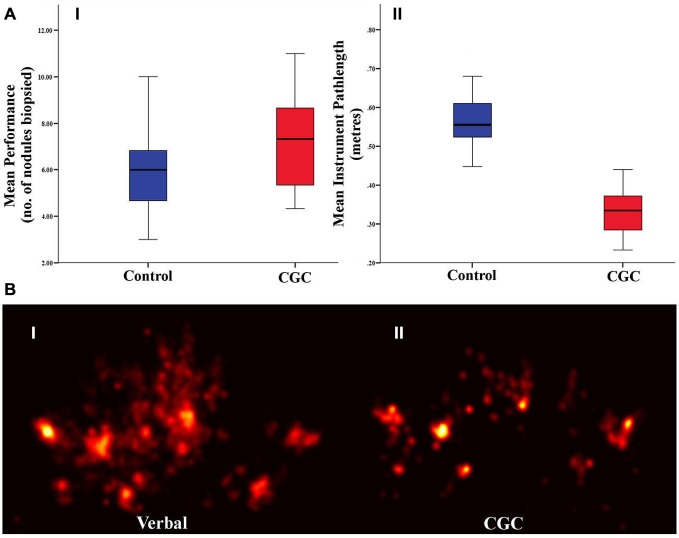
**(A)** Technical performance as indexed by the number of biopsies retrieved (I) and instrument path length (II). Box plots indicate mean and error bars represent 95% confidence interval. **(B)** Gaze plots from a representative subject under control (I) and gaze guidance (II) demonstrate more focussed fixations during gaze-assistance.

Biopsy number and instrument pathlength was analyzed to determine whether CGC improved trainees’ technical performance. As illustrated in Figure 2A, gaze-guidance under the influence of CGC resulted in enhanced technical performance. Table 1 highlights the differences in technical performance according to the mode of guidance. With gaze-assistance, trainees’ biopsied a significantly greater number of nodules [biopsy number (mean ± SD): control = 5.6 ± 1.8 vs. CGC = 6.6 ± 2.0, *p* < 0.05] using significantly shorter instrument pathlength (metres) [mean ± SD: control = 0.6 ± 0.1 vs. CGC = 0.3 ± 0.7, *p* < 0.001]. This implies that trainees were faster, more productive and used virtual instruments more economically when operating from the CGC mode.

**Table 1:** **The influence of guidance mode on technical performance, visual search behavior, changes in cortical hemodynamics, network topological properties and systemic effects**.

Outcome variable	Control condition (Mean ± SD)	CGC condition (Mean ± SD)	*t*-value	*p*-value
**Technical lerformance**
Biopsy number	5.6 ± 1.8	6.6 ± 2.0	−3.394	**0.003**
Instrument path length (m)	0.6 ± 0.1	0.3 ± 0.7	11.765	***0.000***
**Visual search**
Gaze latency (s)	1.4 ± 0.3	0.8 ± 0.2	7.292	***0.000***

**Outcome variable**	**Control condition median (IQR)**	**CGC condition median (IQR)**	***z*-value**	***p*-value**

**Cortical hemodynamics**
ΔHbO_2_ (mMol × cm)	2.5 (12.0)	0.6 (11.2)	−4.049	***0.000***
ΔHHb (mMol × cm)	−1.4 (5.0)	−1.0 (4.5)	−1.098	0.272
ΔHbT (mMol × cm)	3.6 (13)	1.1 (11.6)	−6.064	***0.000***
**Cortical network**
Normalized cost (a.u.)	0.10 (0.13)	0.19 (0.43)	−0.722	0.470
Global efficiency (a.u.)	0.03 (0.05)	0.02 (0.08)	−0.220	0.826
Network burden (a.u.)	0.09 (0.14)	0.18 (0.46)	−0.847	0.397
Network edges (a.u.)	56.0 (304.0)	81.0 (120.0)	−0.589	0.556
**Systemic effect**
Heart rate (beatsmin^-1^)	71.2 (10.0)	73.4 (8.1)	−0.392	0.695
SD_NN_	57.7 (42.0)	47.2 (36.9)	−0.784	0.433

*p < 0.05 = bold, p < 0.001 = bold italic*.

### Gaze Behavior

GL which represents the temporal delay between trainer and trainee gaze fixation was analyzed to determine whether gaze guidance streamlined trainee visual search. Figure 2B depicts the visual search pattern acquired from a representative trainee under both guidance conditions. It is apparent that whilst operating under gaze guidance, trainee fixations appear to be more localized to the nodule to be biopsied. GL was significantly shorter in CGC mode [GL seconds (mean ± SD): control = 1.4 ± 0.3 vs. CGC = 0.8 ± 0.2, *p* < 0.001]. This suggests that gaze assistance manifests as more rapid fixation on the appropriate target nodule to be biopsied.

### Cortical Activation

**Figure 3:**
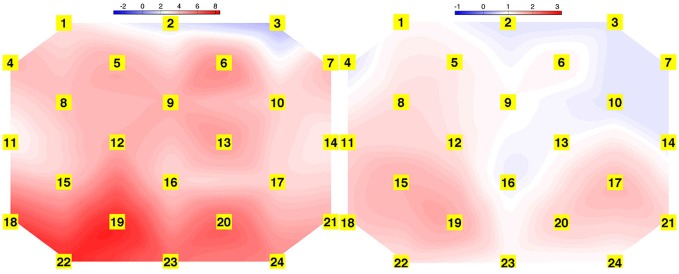
**Topograms derived from task averaged HbO_2_ response of a representative subject for verbal (left) and gaze guidance (right) conditions, depicting spatially broader task-evoked oxygenated hemoglobin change during verbal guidance**.

**Figure 4:**
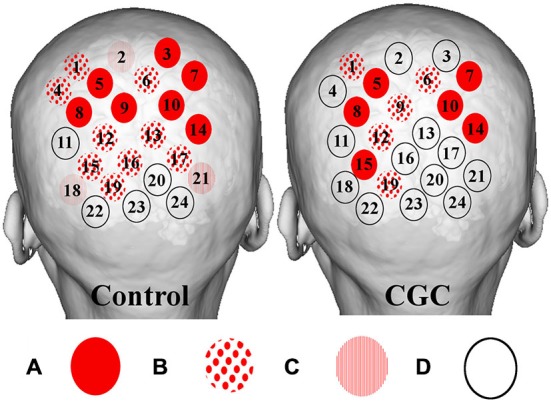
**Figure depicting group averaged (O-P) channel activation for verbal (left) and gaze guidance (right).** Magnitude of statistical changes in cortical hemodynamics reflect intensity of brain activation as follows: **(A)** statistically significant (*p* < 0.05) increase in HbO_2_ coupled to statistically significant (*p* < 0.05) decrease in HHb (red circles); **(B)** increase HbO_2_ and decrease HHb with one species reaching statistical significance, *p* < 0.05 (spots); **(C)** increase HbO_2_ and decrease HHb with neither species reaching statistical significance (stripes); and **(D)** no coupled increase HbO_2_ and decrease HHb (clear circles). Verbal guidance resulted in a greater number of activating channels (control vs. CGC = 19/24 vs. 11/24).

Cortical hemodynamic change was analyzed to compare trainee brain responses between verbal and gaze-assisted modes of operation, with the hypothesis that verbal guidance would induce higher amplitude and spatially broader O-P hemodynamic changes. Topograms of a representative subject depicting the average change in HbO_2_ overlying the O-P cortices are displayed in Figure 3. Table 1, depicts cortical hemodynamic change as ΔHbO_2_ (mMol × cm) averaged across the O-P cortices for both verbal and gaze-guidance. Cortical hemodynamic change evoked by verbal guidance was more diffuse as illustrated in Figure 4 (CGC: 11/24 channels active vs. verbal: 19/24 channels active), more likely to involve bilateral parietal as well as bilateral visual cortices and was greater in magnitude than the response evoked by gaze guidance (ΔHbO_2_ mMol × cm [median (IQR)]: control = 2.5 (12.0) vs. CGC = 0.63 (11.2), *p* < 0.001; ΔHbT mMol × cm [median (IQR)]: control = 3.6. (13.0) vs. CGC = 1.1 (11.6), *p* < 0.001). Overall, this data supports the primary hypothesis that training in CGC mode evokes an attenuated O-P brain response. The mode of guidance did not significantly influence the magnitude of ΔHHb [ΔHHb mMol × cm [median (IQR)]: control = −1.4 (5.0) vs. CGC = −1.0 (4.5), *p* = 0.27]. Similarly, as highlighted in Table 2, REM analysis revealed that guidance mode was a predictor of ΔHbO_2_ (*p* < 0.001) but not of ΔHHb (*p* = 0.19).

**Table 2:** **Results of univariate random effect models (REM), evaluating the influence of the independent variable (mode of guidance) on dependent variables including performance, changes in cortical hemodynamics, cortical network metrics, heart rate (HR) and heart rate variability (HRV)**.

Dependent variable	Coefficient	*S.E.*	*p* > z	95% *C.I.*
Biopsy number	0.090	0.040	**0.025**	0.011 to −0.168
Instrument pathlength (m)	−3.20	0.312	*0.000*	−3.808 to −2.586
Gaze latency (s)	−0.761	0.118	*0.000*	−0.992 to −0.530
ΔHbO_2_ (mMol × cm)	−1.294	0.326	*0.000*	−1.933 to −0.654
ΔHHb (mMol × cm)	−0.198	0.151	0.188	−0.494 to −0.097
ΔHbT (mMol × cm)	−1.094	0.303	*0.000*	−1.689 to −0.500
No. of connections	−0.000	0.000	0.754	−0.002 to −0.001
Normalized cost (a.u.)	−0.036	0.101	0.720	−0.234 to −0.161
Network burden (a.u.)	−0.333	0.097	0.732	−0.244 to −0.157
Global efficiency (a.u.)	−0.106	0.282	0.706	−0.659 to −0.446
Mean HR (beatsmin^-1^)	0.008	0.009	0.353	−0.009 to −0.025
SD_NN_	−0.002	0.003	0.595	−0.008 to −0.004

*(p < 0.05 = bold, p < 0.001 = bold italic)*.

### Cortical Networks

**Figure 5:**
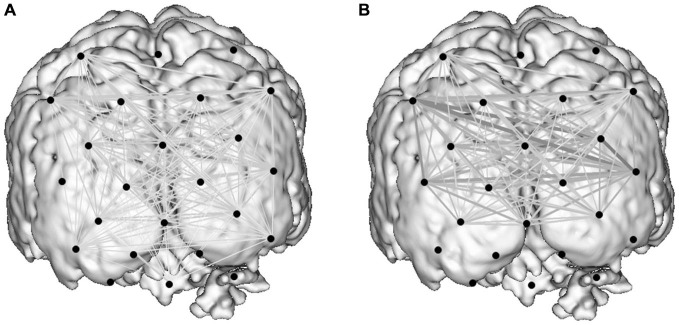
**Activity-guided cortical networks for a representative subject during the control condition (A) and study condition (B).** Approximate channel locations (black circles) are overlain onto reference MRI atlas. The strength of functional associations between nodes in the network is represented by the boldness of network edges.

Graph theoretical econometric data were computed and compared between guidance modes with the hypothesis that the performance of functional network in CGC mode would be associated with less cost and greater efficiency. Figure 5 depicts the activated cortical network under control and CGC conditions for a representative subject. Table 1 represent results of econometric analysis delineating the number of cortical connections, normalized cost, maximum global efficiency and cognitive burden. Differences in these network topological properties between modes guidance did not reach statistical threshold. Additionally, even when subject-level clustering was considered (Table 2) guidance mode was not found to predict network properties (e.g., cost, efficiency, etc). This suggests that CGC does not induce changes in the trainee’s O-P functional network observable with the current OT technique.

### Heart Rate Data

HR and SD_RR_ were monitored to determine the influence of guidance mode on stress-related change in systemic responses (Table 1). Between-condition differences in HR and SD_RR_ were not statistically significant [Median HR (IQR): control = 71.2 (10.0) vs. CGC = 73.4 (8.1) *p* = 0.70; Median SD_RR_ (IQR): control = 57.7 (42.0) vs. CGC = 47.2 (36.9), *p* = 0.43). Additionally, upon REM analysis, neither HR nor SD_RR_ were observed to be predictors for changes in cortical hemodynamics.

### Harms

No harms occurred in the study.

## Discussion

In this study, performance on a simulated surgical task has been improved by modulating the manner in which collaborating surgeons interact with one another. Communicating through collaborative gaze-driven control leads to a greater number of successful biopsies and a reduction in instrument path length, the latter being a measure of dexterity previously shown to reflect skill level in laparoscopic and open surgery (Bann et al., 2003; Xeroulis et al., 2009). The foundation for this improvement appears to be a change in visual search strategy manifest as a reduced GL indicating that with gaze-assistance, trainee fixation points more rapidly reach those of the expert. This was accompanied by an amelioration of cortical excitation across primary visual centers in the brain, but without an appreciable difference in O-P network costs or burden.

The current paper offers a potential mechanistic explanation for improvements observed in novices’ performance when training under the influence of expert visual cues (Wilson et al., 2011; Chetwood et al., 2012). Experienced operators are known to utilize more effective gaze-strategies than novices, characterized by fixating on relevant target locations and adopting optimal psychomotor control (Wilson et al., 2011). Unlike novices who learn mapping rules by switching their point of regard between tool and target, experts utilize a target locking strategy and rarely need to check tool locations (Leong et al., 2008). As demonstrated by Wilson et al. (2011), novices trained to observe and then “mimic” the more focused gaze patterns of experts improve their laparoscopic performance and multi-tasking capabilities more than novices trained to observe expert performance without the benefit of expert gaze-cues. Similarly, Chetwood et al. (2012) observed improved completion times and reduced errors in novices guided by expert gaze vs. expert verbal instructions. However, unlike the current experiment, the aforementioned studies were not designed to explain the foundation for improved performance owing to gaze guidance, resulting instead in speculation regarding adaptation in visual cognitive function. Here, improved performance as a result of expert gaze guidance is understood as a reduction in visual activation and hence attentional demand on the visual cortex. This is in line with studies demonstrating learning related plasticity in activation maps implying attenuation of attentional resources associated with training and expertise (Dayan and Cohen, 2011). By manipulating the visual behavior of novices in a way that they align more closely with those of experts it is conceivable that novices may bypass the early “cognitive” phases of visual-motor learning (Fitts and Posner, 1967). This notwithstanding confirming that the gaze behavior of trainees operating under gaze guidance was characterized by less random saccadic activity and was indeed more “expert” cannot be confirmed using GL alone and would necessitate a more elaborate analysis of eye-tracking data such as using exploit/explore ratio (Dehais et al., 2015) or visual entropy (Di Nocera et al., 2007).

There is evidence from functional neuroimaging studies that streamlined visual search strategies lead to reduced activation in the visual cortex (Kojima and Suzuki, 2010). For example, Kojima and Suzuki (2010) observed greater hemodynamic responses in fNIRS channels centered on the visual cortex during more effortful search strategies. However, it must be acknowledged that the introduction of a target feature into the surgical scene might be anticipated to increase visual attention owing to changes in visual saliency. This is relevant since the eye-tracking derived fixation point of the expert was projected to trainee as a visually salient target. Interestingly, shifts in visual attention secondary to manipulations in visual saliency as a result of gaze-guidance (i.e., the trainer’s fixation point) did not manifest as greater activation in the visual cortex when compared to verbal instruction. Rather, the resultant visual search is potentially streamlined from a *“top-down”* to *“bottom-up”* strategy (van der Stigchel et al., 2009; Theeuwes, 2010). Specifically, if a target markedly differs from its background, it is visually salient and is more likely to be detected by a *“bottom-up”* search strategy guided by the saliency of the scene, whereas if a target requires greater cognitive input to be identified, a *“top down”* search ensues which is dependent on the PFC and parietal cortex (PC; van der Stigchel et al., 2009; Theeuwes, 2010). Bottom up saliency is not coded in the primary visual cortex (Betz et al., 2013), and this mode results in search simplification leading to a reduction in activity in visual association areas (Kojima and Suzuki, 2010). Enhanced saliency through visual guidance may parallel visual processing of natural stimuli (Einhäuser and König, 2010), whereby responses in V1 cells are optimally sparse (Vinje and Gallant, 2000). In the current study, this effect has been observed as a reduction in O-P cortical hemodynamic changes with comparatively fewer channels reaching statistical threshold for activation.

Parietal cortical activity is also associated with oculomotor intention and attention and may be important in planning eye movements (Kanwisher and Wojciulik, 2000). Verbal guidance may result in demanding visual search since it necessitates that auditory information be explicitly processed and translated into visual-spatial co-ordinates to understand the desired target’s location, and parietal lobe activation has been shown to be important in spatial integration (Molholm et al., 2006). Conversely, gaze-guidance protocols may share many similarities with implicit learning protocols (Wilson et al., 2011). Implicit learning, a form of unconscious, incidental and procedural knowledge demands fewer attentional resources than explicit learning, a form of conscious, intentional or declarative knowledge. Implicit motor learning has been shown to reduce non-essential co-activation or connectivity between verbal-analytic and motor planning regions during laparoscopic performance (Zhu et al., 2011).

Here, as well as investigating connectivity (i.e., correlations), network topology has been explored with graph theory, which provides a powerful method for quantitatively describing the topology of brain connectivity (He and Evans, 2010). Graph theory has been utilized to interrogate cortical networks in both pathological and non-pathological brains (Achard and Bullmore, 2007; Bassett et al., 2009), and allows network parameters such as cost and efficiency to be determined (Bullmore and Bassett, 2011). Presently, graph theory was applied to experimental data in order to further appreciate the impact of a “gaze-channel” on functional brain networks. From the active network analysis (i.e., that which retains only activated nodes), it is evident that compared to verbal-guidance, gaze-assistance does not lead to significant differences in O-P network topologies, therefore disproving the secondary hypothesis. Therefore, our conclusion is that collaborative gaze exerts a positive effect on technical skills, alleviates burden on the visual cortices, and yet critically does not significantly alter performance of the functional O-P network.

Intuitively verbal instructions about target location are time consuming to deliver, more complex to interpret and harder to translate into the “visual” workspace, ultimately relying therefore on greater cognitive work as evidenced by enhanced task performance when visual guidance is employed (Chetwood et al., 2012). We suspect that gaze assistance makes the flow of information between the trainer and trainee more seamless by increasing the perceptual fidelity of the instruction given. Extrapolating this effect to the *in vivo* setting, a reduction in the attentional demands necessary to execute a procedure may manifest as a liberation of resources to devote to other safety critical aspects of clinical care (e.g., reacting to unexpected events, multitask decision making, planning operative steps, *etc*.). Future studies may capitalize on a framework that enables combined analysis of brain responses, visual behavior and HRV to improve the detection of changes in workload as has been demonstrated in pilots (Duratin et al., 2014). Furthermore, although not specifically investigated within the confines of this study, it is feasible that in using visual guidance the need to verbalize the intended target is bypassed and as such the trainer can focus on supplementary aspects of the procedure. For example, if the site of suture placement is already determined and displayed visually, a trainer can then focus verbal instruction on the technical aspects of suturing manoeuvres required to achieve accurate tissue apposition.

## Conclusion

To summarize, this study demonstrates that capitalizing on visual behavior enhances communication between collaborating surgeons, and improves operator performance. This may be achieved through a *bottom up* allocation of resources within the visual cortex of the surgeon being instructed. It is plausible that trainees instructed in this fashion will be better able to devote neural resources to other safety critical aspects of the procedure. In investigating these hypotheses, fNIRS technology is well placed to make an impact, as it overcomes the limitations of traditional scanning environments (Cutini et al., 2012). However, future validation of graph theory measures for fNIRS connectivity analysis will necessitate comparison against models of anticipated responses and structural connectivity as have been observed using other neuroimaging technologies such as fMRI (van den Heuvel et al., 2009; Zhang et al., 2010). Critically, demonstration of correspondence between predicted and observed patterns of functional connectivity would support the feasibility and validity of fNIRS-derived connectivity measures.

## Author Contributions

Study design and protocols were conceived by DRCJ, DRL, FO-E, LWS, K-WK, GM, G-ZY, and AWD. Data collection was performed by DRCJ, DRL, FO-E, LWS and K-WK. Data analysis was performed by DRCJ, FO-E, DRL, K-WK, LWS, GM and TA. The manuscript was written by DRCJ, DRL and FO-E and final critical editing was performed by DRL, LWS, GM, TA, G-ZY and AWD.

## Funding

This work was funded in part by research grants from the Academy of Medical Sciences (Lecturer Starter Grant) and Cancer Research UK (Academic Lecturership).

## Conflict of Interest Statement

The authors declare that the research was conducted in the absence of any commercial or financial relationships that could be construed as a potential conflict of interest.
